# Hydrogen Peroxide as a Disinfectant and Germicide

**Published:** 1888-03

**Authors:** W. D. Bizzell

**Affiliations:** Professor of Principles and Practice of Medicine in the Southern Medical College, Atlanta, Georgia


### HYDROGEN PEROXIDE AS A DISINFECTANT AND GERMICIDE.

BY W. D. BIZZELL, M. D.,

Professor of Principles and Practice of Medicine in the Southern Medical College, Atlanta, Georgia.

Peroxide of hydrogen was first discovered by Thenard, the French chemist, 1818, and in recent years has been brought to the attention of the medical profession by Dr. B. W. Richardson, of London.

Its chemical composition, as is well known, is $H_2 O_2$ or ($H_2$ O or water + O); as water is a saturated molecule, the oxygen in the peroxide is held very loosely and is very easily released from its anomalous situation, chemically speaking. Oxygen, when so liberated in the nascent or new-born condition, possesses, as is well known, extraordinary chemism or affinity and will seize on and unite with the constitutent atoms of any molecule within reach, except those of the simplest and most stable character. The pure $H_2 O_2$ of syrupy consistence will attack living as well as dead structure possessing true caustic properties. But dissolved in water, even when in a stronger solution than is necessary to disinfect the most putrid wound or pus cavity, is entirely harmless, and scarcely more irritating than distilled water.

The obstacle in the way of a more extended use of this really remarkable agent is the difficulty in obtaining a watery solution that is of reliable strength, and of preventing a rapid deterioration after it is obtained. The strong solution in water looks like water, is without odor, and has only the faintest, pungent, slightly metallic taste, not more so than many specimens of water obtained from metallic pipes or vessels. The only practical test that I have found necessary is to add a small quantity to stale urine, egg albumen and water, or to drop a little on pus or other discharge from wounds, and watch the rapid evolution of the oxygen gas ; when applied to open wounds or injected into cavities, it gives rise to this rapid bubbling and effervesence of gas, destroying odor, and in the case of cavities producing quite a rapid regurgitation of a frothy character. If this result does not follow, or is only feebly expressed, you may be sure that either you are using a solution much too dilute or the preparation has deteriorated and is no longer of any value.

Experiments were made by Dr. P. Miguel as to the quantity of various substances, commonly used as germicides, which, added to a quart of beef-tea, would prevent decomposition.

He found, among a long list of substances used, only two more powerful than $H_2$ $O_2$, viz.:

| | |
|---|---|
| Biniodide mercury . . . . . . . . . . | 0.025 grams. |
| Biniodide silver . . . . . . . . . . . | 0.03 grams. |
| Hydrogen Peroxide . . . . . . . . | 0.05 grams. |
| Bichloride mercury . . . . . . . . | 0.07 grams. |

It will thus be seen that, properly diluted, we have a bland, unirritating and non-poisonous disinfectant and germicide that, on account of its gaseous nature, will diffuse itself into every pocket, nook or cranny of a fistulous tract or abscess cavity. Where we are not sure of immediate drainage, or where to irrigate or reach every part of a suppurating cavity is difficult or impossible, the peroxide of hydrogen is undoubtedly of inestimable value.

On account of first cost and the difficulty of preventing deterioration, as it should be kept from the light, tightly corked, and at a temperature below 70 degrees F., if possible, $H_2$ $O_2$ will necessarily be only used in selected cases.

Those who have treated suppurative inflammation of the antrum will recall how difficult it is to cure these cases even after drilling through the floor so as to more readily command the cavity. In these cases, if there be no dead bone, fangs, periostitis, etc., we can safely promise a rapid and permanent cure. The following case serves to illustrate:

A. B., white, aged 36, consulted me for what he supposed was catarrh or neuralgia, or both. Complained of a distended feeling in the right side of the face with pains in the brow, cheek, orbit and upper teeth; at times quite an offensive discharge from the nose. Said that a dentist had removed a second molar tooth from the upper jaw of this side. The tooth was badly decayed and there was an abscess at the root. On examination found this cavity had not healed, but was filled with unhealthy granulations, through which, by careful manipulation, I passed a probe into the antrum, giving exit to a few drops of very offensive pus.

Carried him over to Dr. Catchings, who, with his dental engine, enlarged the opening so as to command the cavity. I then placed him in his hands for treatment. Daily injections of carbolized water, chloride of zinc solutions, etc., were used, but the tendency to pus-formation continued. After three or four weeks' treatment, the doctor left the city for a few days and the patient returned to me. I injected a ten per cent. solution of $H_2 O_2$. In a very short time the evolution of oxygen caused a frothing through the artificial opening, alveolar ridge, and also through the nasal opening of the antrum into the nose and throat, bringing with it all of the pus and other secretions of the antrum whitened, oxydized and rendered entirely innoxious. The opening in the floor of the antrum was then tightly plugged with iodoform cotton. A repetition of this procedure for several days in succession and a careful plugging of the opening was all that was necessary to effect a perfect cure.

CASE II.—SCROFULOUS ABSCESS OF THE NECK AND FACE.

C. A., negro child, aged nine years. The whole side of the neck and face was infiltrated with pus and outlying œdema. Eye on right side almost entirely closed. No heat of surface. Made an incision over the most prominent point near angle of the jaw, giving exit to an immense quantity of horribly offensive pus. Injected ten per cent. solution $H_2 O_2$. All fetor immediately disappeared. A repetition of this injection for a few days, and the internal administration of syr. ferri iodidi was soon followed by a good recovery.

CASE III.—PERINEPHRITIC ABSCESS.—Mrs. S. J., white, aged about 35. History of pain in the side for six weeks past; been confined to bed for three weeks; supposed caused a jar or strain from falling off a fence and landing on the feet. On examination found a swelling in lumbar region, with greatest prominence one and a half inches above the crest of the illium. The bogginess and sense of flatuation convinced me there was pus rather deeply placed; introduced fine needle of hypodermic and aspirated a few drops of pus; took a curved bistoury and at a depth of two and a half inches opened the pus cavity. During the next twenty-four hours there drained out fully a quart of pus. Becoming convinced that the drainage was not sufficiently free, passed in a drainage tube fourteen inches in length. On account of the extent of the pus cavity, its relations to loose connective tissue, and the liability to retain such materials as might be injected into it, I deemed it unwise to use solutions of bichloride mercury, carbolic acid or any substance likely to produce toxic effect from absorption. Used a seven per cent. solution of hydrogen peroxide in warm water, injecting it deep into the bottom of the cavity with long-nozzle, hard-rubber syringe. This was repeated every day, the tube shortened one inch daily, and a pad of iodoform absorbent cotton laid over the wound. The patient improved rapidly, and the tube was removed on the ninth day, leaving only a shallow fistula where the tube had rested, and this quickly healed.

